# The Association of *VDR*, *CYP2R1*, and *GC* Gene Polymorphisms, Dietary Intake, and BMI in Regulating Vitamin D Status

**DOI:** 10.3390/diseases13070219

**Published:** 2025-07-14

**Authors:** Carmina Mariana Stroia, Annamaria Pallag, Maria Vrânceanu, David de Lorenzo, Keith Anthony Grimaldi, Csaba Robert Pallag, Kinga Vindis, Diana Bei, Cristina Burlou-Nagy (Fati), Timea Claudia Ghitea

**Affiliations:** 1 Doctoral School of Biomedical Sciences, Faculty of Medicine and Pharmacy, University of Oradea, 410073 Oradea, Romania; stroia.carminamariana@student.uoradea.ro (C.M.S.); vindis.kinga@student.uoradea.ro (K.V.); burlounagy.cristina@student.uoradea.ro (C.B.-N.); 2 Pharmacy Department, Faculty of Medicine and Pharmacy, University of Oradea, 410073 Oradea, Romania; timea.ghitea@csud.uoradea.ro; 3 Department Nutrition and Dietetics, Faculty of Food Engineering, Tourism and Environmental Protection, Aurel Vlaicu University of Arad, 310025 Arad, Romania; marievranceanu@gmail.com; 4 UCL Great Ormond Street Institute of Child Health, London WC1N 1EH, UK; david.delorenzo@ucl.ac.uk; 5 Department of Nutrigenetics and Personalized Nutrition, Eurogenetica, 00185 Rome, Italy; keith.grimaldi@gmail.com; 6 MSc International Economy and Business Program of Study, Corvinus University of Budapest, 1093 Budapest, Hungary; csaba.pallag@stud.uni-corvinus.hu; 7 Medical Disciplines Department, Faculty of Medicine and Pharmacy, University of Oradea, 410073 Oradea, Romania; diana.bei@didactic.uoradea.ro

**Keywords:** vitamin D, BMI, genetic polymorphisms, *VDR*, *CYP2R1*, *GC*, 25(OH)D, dietary intake

## Abstract

Vitamin D plays a crucial role in bone health and immune function, with serum 25(OH)D levels influenced by genetic, dietary, and metabolic factors. Background/Objectives: This study investigated the impact of *VDR* rs731236, *CYP2R1* rs10741657, and *GC* rs2282679 polymorphisms, body mass index (BMI), and dietary vitamin D intake on vitamin D status. Methods: A total of 230 adults were classified into four BMI categories: normal weight (NW), overweight (OW), obesity class I (OB), and obesity class II/III (OP). Participants completed a Food Frequency Questionnaire (FFQ) and a 7-day Food Frequency Diary (FFD). Genotyping was performed using TaqMan assays, and serum 25(OH)D was quantified via spectrophotometry. Statistical analyses included ANOVA and multiple linear regression. Results: The *VDR* rs731236 CC genotype, *CYP2R1* rs10741657 AG/GG, and *GC* rs2282679 AC/CC were associated with lower serum vitamin D levels. A higher BMI was significantly correlated with reduced serum 25(OH)D (*p* < 0.001), with BMI emerging as the strongest predictor of vitamin D status. FFQ-based dietary intake showed a modest positive correlation with 25(OH)D (r = 0.47, *p* < 0.001). Conclusions: BMI and genetic variants in *VDR*, *CYP2R1*, and *GC* significantly influence vitamin D metabolism. Personalized interventions addressing genetic predispositions and weight management may improve vitamin D status.

## 1. Introduction

Vitamin D insufficiency is a widespread global health issue, with an estimated prevalence of 15.7% between 2000 and 2022 [1]. In Romania, comprehensive data are lacking, however, a 2015 study reported that up to 59% of individuals—especially older adults, women, and those during winter—exhibit poor vitamin D levels [2]. In response, the Ministry of Health initiated a national program for vitamin D status evaluation in high-risk groups. According to the program’s 2022 report, 39.83% of adults had a vitamin D deficiency (<20 ng/mL), despite 13.4% of them taking supplements [3]. Severe vitamin D deficiency results in osteomalacia, osteoporosis, and fracture [4,5], but it is also associated with non-skeletal disorders such as cardiovascular diseases [6] obesity [7], diabetes [8], asthma [9], and multiple sclerosis [10], in addition to its well-established function in skeletal health. It is well established that over 90% of the vitamin D required for human health is synthesized endogenously via cutaneous production stimulated by ultraviolet B (UVB) radiation, particularly within the 290–315 nm spectrum. In European populations, especially in Western countries, 80–90% of vitamin D is estimated to derive from such endogenous synthesis, depending on latitude, season, and lifestyle [11]. Vitamin D3 is derived from animal products such as fish, meat, and milk [12], while vitamin D2 comes from certain mushrooms, yeast, fortified foods, cocoa butter, and cocoa powder [13]. Although vitamin D3 is considered more bioavailable than vitamin D2 [14], both undergo similar metabolic pathways [15].

Vitamin D metabolism involves a complex enzymatic pathway including genes such as *DHCR7*, *CYP2R1*, *CYP27B1* [16], *CYP24A1*, and *GC*, which together govern the synthesis, activation, transport, and degradation of vitamin D metabolites [17].

Among these, we focused on three well-characterized single nucleotide polymorphisms (SNPs)—*VDR* rs731236, *CYP2R1* rs10741657, and *GC* rs2282679—selected based on functional relevance, reproducibility in population-based cohorts, and replicated associations in vitamin D studies [18]. These variants have been associated with serum 25-hydroxyvitamin D [25(OH)D] concentrations across diverse populations, highlighting their central role in interindividual variability of vitamin D status [17,19,20,21,22,23,24,25].

In this context, the findings from this study will contribute to a more comprehensive understanding of how genetic and environmental factors interact in vitamin D metabolism. Despite the well-documented inverse relationship between BMI and serum 25(OH)D levels, clinical studies suggest that weight loss often leads to only modest improvements in vitamin D status. This supports the hypothesis that mechanisms such as volumetric dilution and adipose tissue sequestration, rather than decreased intake or malabsorption alone, play a central role in obesity-related hypovitaminosis D [22].

The findings from this study will contribute to a more comprehensive understanding of how genetic and environmental factors interact in vitamin D metabolism, providing valuable insights for future public health strategies in Romania [26].

The main hypothesis of this study is that both BMI and genetic polymorphisms (*VDR* rs731236, *CYP2R1* rs10741657, and *GC* rs2282679) significantly influence serum 25(OH)D levels, providing insights into the interplay between genetic and environmental factors in vitamin D metabolism.

## 2. Materials and Methods

This study was conducted between 1 October 2024 and 30 November 2024, following approval from the Research Ethics Committee of the Faculty of Medicine and Pharmacy, University of Oradea (Decision No. CEFMF/2 of 29 July 2022) and the Nutrition Clinique—CSBNDIET PRECISION NUTRITION SRL (Decision No. 7/30 September 2024). All investigations adhered to the principles of the Declaration of Helsinki and the Romanian Code of Medical Deontology. Written informed consent was obtained from all participants, and all data collection complied with General Data Protection Regulation (GDPR) requirements.

Participants were recruited and monitored through Nutrition Clinique—CSBNDIET PRECISION NUTRITION SRL, a private practice specializing in personalized nutrition-based interventions, located in Târnăveni, Romania.

Inclusion criteria were as follows: adults aged ≥ 18 years, in apparent good health, without known chronic conditions or bone metabolism disorders at the time of data collection, not currently taking medications known to interfere with vitamin D metabolism, and without vitamin D supplementation in the previous six months.

Exclusion criteria included the following: a medical history of chronic diseases (e.g., liver or renal disorders, endocrine or autoimmune conditions, malabsorption syndromes), any diagnosed bone-related pathology (e.g., osteoporosis, osteomalacia, Paget’s disease), pharmacological treatments affecting vitamin D pathways (e.g., glucocorticoids, anticonvulsants), pregnancy, or lactation.

### 2.1. Study Population

A total of 230 individuals participated in this study, categorized into four groups: NW—normal weight *(n* = 50, BMI 18.5–24.9 kg/m^2^), OW—overweight (*n* = 59, BMI 25–29.9 kg/m^2^), OB—obesity class I (*n* = 62, BMI 30–34.9 kg/m^2^), and OP- obesity class II/III (*n* = 59, BMI ≥ 35 kg/m^2^).

This study assessed genetic polymorphisms in the *VDR*, *GC*, and *CYP2R1* genes, along with serum 25(OH)D levels. Upon enrollment, participants attended a single visit to the Nutrition Clinic, where oral swabs were collected for DNA extraction and blood samples were taken for serum 25(OH)D measurement. Saliva samples were sent to the genetic laboratory at NKAARCO Diagnostics Limited (Norwich, UK), and blood samples were processed by a certified clinical laboratory for 25(OH)D quantification.

### 2.2. Dietary Assessment

Vitamin D intake was evaluated using two complementary tools: a Food Frequency Questionnaire (FFQ) and a 14-day Food Frequency Diary (FFD), in order to capture both habitual patterns and short-term dietary variation. The FFQ, adapted from the validated Food4Me tool, allowed for a retrospective estimation of vitamin D intake, but is known to be prone to overreporting due to recall bias [27]. In contrast, the FFD provided more precise, day-to-day intake data, but has been associated with underreporting, particularly in free-living individuals. Dietary data from both instruments were manually processed using Microsoft Excel. Vitamin D content was calculated based on standard portion sizes and food composition values from two harmonized sources: the EFSA Food Composition Database and the USDA National Nutrient Database. This approach ensured consistency across all micronutrient estimates. To enhance accuracy and reduce method-related bias, we applied an integrative strategy for estimating total vitamin D intake (IU/day). Specifically, a harmonized value was calculated as the b value, reflecting the midpoint between potential overreporting (FFQ) and underreporting (FFD). This correction strategy has been previously used to improve comparability between dietary assessment methods. To validate this combined approach, a Bland–Altman analysis was performed, revealing a mean difference of +35.12 IU/day (FFQ − FFD), with 95% limits of agreement from −254.03 to +324.26 IU/day. These findings indicate acceptable agreement for use in population-level estimates of vitamin D intake [28,29,30,31,32].

### 2.3. Genotyping and Data Collection

Buccal swab samples were collected from all participants using the Isohelix^®^ Buccalyse DNA Extraction Kit BEK-50 (Isohelix^®^, Kent, UK), following the manufacturer’s instructions. Genomic DNA was extracted and quantified via spectrophotometry to ensure appropriate concentration and purity.

Genotyping was performed for three single nucleotide polymorphisms (SNPs) involved in vitamin D metabolism: VDR rs731236 (T > C), CYP2R1 rs10741657 (A > G), and GC rs2282679 (A > C). Samples were shipped to NKAARCO Diagnostics Limited (Norwich, UK), where real-time PCR genotyping was conducted using TaqMan SNP Genotyping Assays (Thermo Fisher Scientific, Waltham, MA, USA) on an ABI7900HT or StepOnePlus Real-Time PCR System (Applied Biosystems, Foster City, CA, USA).

The PCR protocol was standardized: initial denaturation at 95 °C for 10 min, followed by 40 cycles of denaturation at 95 °C for 15 s, and annealing/extension at 60 °C for 60 s. Genotyping was performed in 10 µL reaction volumes containing 5 µL of TaqMan Genotyping Master Mix, 0.5 µL of SNP Genotyping Assay Mix (specific assay IDs: VDR rs731236: C__12060045_20, CYP2R1 rs10741657: C__3209265_10, GC rs2282679: C__8278879_10), 10 ng of genomic DNA, and nuclease-free water. Allelic discrimination was carried out using Sequence Detection Systems (SDS) software, version 2.2.1 (Applied Biosystems).

### 2.4. Biochemical Markers

The primary biochemical marker analyzed was 25(OH)D. Additional serum markers, including C-reactive protein (CRP), were measured using validated clinical assays, and were included in secondary exploratory analyses. Blood samples were collected from the antecubital vein after an overnight fast of 8–12 h, with participants seated during collection. A tourniquet was applied and released prior to venipuncture. Blood was drawn into Vacutainer tubes (Becton Dickinson, Mountain View, CA, USA) containing a clot activator and gel separator for serum separation, following standard procedures [33].

#### 2.4.1. Measurement of 25(OH)D Levels

To assess vitamin D status, serum 25-hydroxyvitamin D [25(OH)D] levels were measured using the ST AIA-PACK 25-OH Vitamin D reagent (Tosoh Bioscience, San Francisco, CA, USA) on a TOSOH 900 spectrophotometer (Tosoh Bioscience, San Francisco, CA, USA). Blood samples were collected after an overnight fast, stored in clot activator tubes, and transported to a certified reference laboratory for analysis. Given its relatively long half-life of approximately 2–3 weeks, 25(OH)D is considered the most reliable biomarker for assessing vitamin D status, reflecting both endogenous synthesis and dietary intake [18,34,35,36,37].

According to established clinical guidelines, vitamin D status was classified as follows: deficient: ≤20 ng/mL; insufficient: 21–29 ng/mL; and optimal: 30–100 ng/mL.

#### 2.4.2. Measurement of High-Sensitivity C-Reactive Protein (hs-CRP)

To assess low-grade systemic inflammation with higher analytical sensitivity, serum high-sensitivity C-reactive protein (hs-CRP) concentrations were measured using an immunoturbidimetric assay on the Atellica CH Analyzer (Siemens Healthineers, Erlangen, Germany), following the manufacturer’s protocol.

The analysis was conducted using the Atellica CH hsCRP Reagent Kit, designed for the quantitative determination of hs-CRP in human serum or plasma.

The assay has a validated measuring range of 0.16–10.0 mg/L, with a limit of detection (LoD) of ≤0.07 mg/L and high analytical concordance with reference methods such as ELISA and nephelometry (>90%).

Serum hs-CRP concentrations were interpreted using clinical thresholds endorsed by the American Heart Association (AHA) and Centers for Disease Control and Prevention (CDC): <1 mg/L: low cardiovascular and metabolic inflammation risk; 1–3 mg/L: intermediate risk; >3 mg/L: high risk; and >10 mg/L: may reflect acute infection or trauma; these thresholds were analyzed separately.

These thresholds have been applied in multiple metabolic and cardiovascular studies. These interpretation thresholds are in line with current clinical guidelines and have been applied in several metabolic and inflammatory studies.

### 2.5. Anthropometric Measurement

Anthropometric measurements were performed in the morning after an 8 h fast, with participants wearing minimal clothing (underwear, no shoes). BMI was calculated as weight in kilograms divided by height in meters squared (kg/m^2^), and classified according to WHO criteria [38] into underweight (BMI < 18.5 kg/m^2^), normal weight (18.5–24.9 kg/m^2^), overweight (25–29.9 kg/m^2^), obesity class I (30–34.9 kg/m^2^), obesity class II (35–39.9 kg/m^2^), and obesity class III (≥40 kg/m^2^).

### 2.6. Statistical Analysis

All statistical analyses were performed using IBM SPSS Statistics version 25.0 (IBM Corp., Armonk, NY, USA) and GraphPad Prism version 9.0 (GraphPad Software, San Diego, CA, USA). Data distribution was assessed using the Shapiro–Wilk test. For continuous variables with normal distribution, intergroup comparisons across BMI categories were evaluated using a one-way ANOVA followed by Bonferroni post hoc correction. For non-normally distributed variables, the Kruskal–Wallis H test was applied.

Associations between continuous variables were assessed using Spearman’s rank correlation coefficient. Genotype–phenotype relationships were analyzed via two complementary approaches: (1) Spearman correlation using ordinal genotype coding (0 = major-allele homozygote, 1 = heterozygote, 2 = minor-allele homozygote); and (2) Kruskal–Wallis analysis across genotype groups.

Multiple linear regression was employed to identify independent predictors of serum 25(OH)D levels. A two-way ANOVA was used to assess potential interaction effects between BMI categories and vitamin D assessment method (serum, FFQ, FFD). Agreement between dietary assessment tools (FFQ vs. FFD) and serum 25(OH)D was evaluated using Bland–Altman analysis.

Bonferroni-adjusted *p*-values were applied to correct for multiple comparisons where appropriate. A two-tailed *p*-value < 0.05 was considered statistically significant.

## 3. Results

### 3.1. Baseline Characteristics

This study included 230 participants, of whom 32.6% were male *(n* = 75) and 67.4% were female (*n* = 155), distributed across four BMI categories. A significant gender imbalance was observed, with a higher proportion of females in all groups. The mean age varied slightly across categories, ranging from 36 years in the normal-weight (NW) group to 39.07 years in the obesity class I (OB) group. The overweight (OW) group had a mean age of 38.89 years, while the obese class II/III (OP) group had a mean of 37 years.

Table 1 summarizes the demographic and anthropometric characteristics of participants across BMI categories, offering contextual information relevant for subsequent analyses.

### 3.2. Genotype Distribution and Allele Frequencies

Genotyping was performed for three key polymorphisms involved in vitamin D metabolism, namely *VDR* rs731236, *CYP2R1* rs10741657, and *GC* rs2282679. As shown in Table 2, the *VDR* rs731236 SNP exhibited a statistically significant distribution (χ^2^ = 7.59, *p* = 0.022), as did *CYP2R1* rs10741657 (χ^2^ = 11.62, *p* = 0.003), while *GC* rs2282679 did not show a significant association (χ^2^ = 2.39, *p* = 0.42).

### 3.3. Genotype-Specific Distributions of BMI

**Figure 1:**
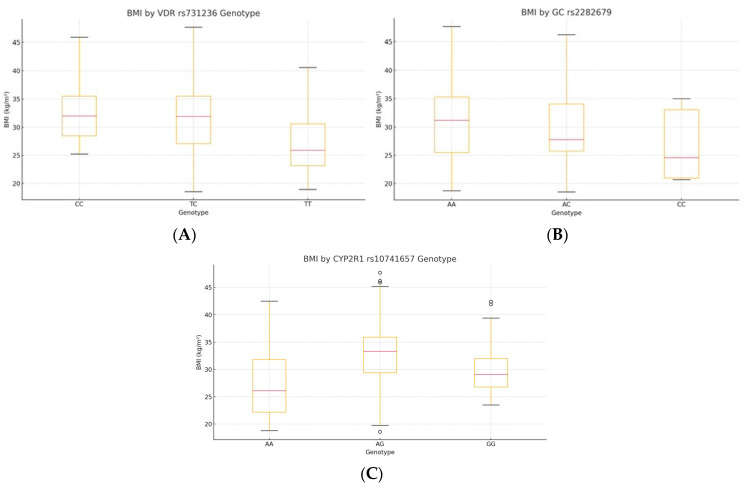
Genotype-specific distributions of BMI: (**A**) VDR rs731236, (**B**) CYP2R1 rs10741657, and (**C**) GC rs2282679. The central line indicates the median; boxes represent the interquartile range (IQR); and whiskers denote the minimum and maximum values within 1.5× IQR. Outliers are shown as individual points where applicable.

Kruskal–Wallis testing showed significant BMI differences by genotype for *VDR* rs731236 (H = 25.45, *p* = 1.2 × 10^−6^, ε^2^ = 0.105) and *CYP2R1* rs10741657 (H = 32.61, *p* = 1.7 × 10^−7^, ε^2^ = 0.137), whereas *GC* rs2282679 had no effect (H = 4.44, *p* = 0.18). Post hoc Bonferroni tests indicated lower BMI in TT carriers of VDR versus TC and CC (all p_adj < 0.01) and a higher BMI in AA carriers of CYP2R1 versus AG and GG (all p_adj < 0.01) (Table 3, Figure 1; see Supplementary Table S1 for pairwise comparisons).

### 3.4. Genotype-Specific 25(OH)D Associations

**Figure 2:**
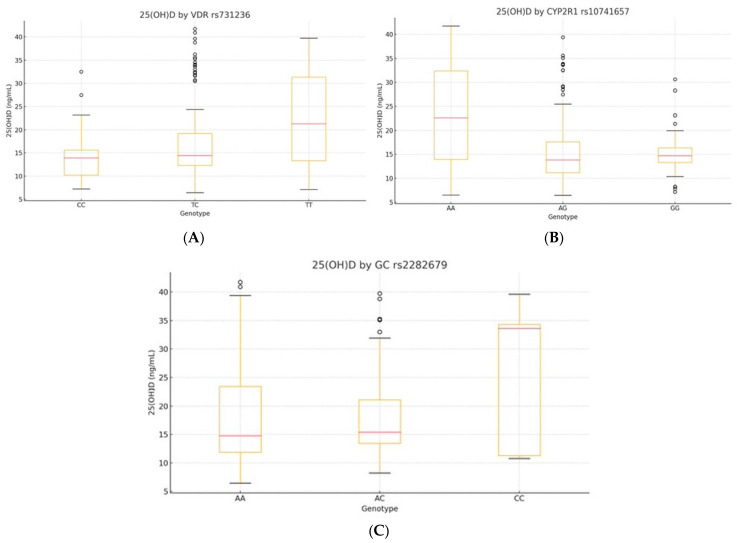
Boxplots of serum 25(OH)D by genotype: (**A**) *VDR* rs731236, (**B**) *CYP2R1* rs10741657, and (**C**) *GC* rs2282679. The central line indicates the median; boxes represent the interquartile range (IQR); and whiskers denote the minimum and maximum values within 1.5× IQR. Outliers are shown as individual points where applicable.

We evaluated serum 25(OH)D levels across genotypes of *VDR* rs731236, *CYP2R1* rs10741657, and *GC* rs2282679 using a one-way ANOVA. Significant differences were observed for *VDR* and *CYP2R1*, but not for *GC* (Table 4, Figure 2A–C; see also Supplementary Table S2 for full model statistics).

*VDR* rs731236 showed a strong association (F(2, 227) = 15.34, *p* < 0.001, η^2^ = 0.12), with TT carriers displaying higher 25(OH)D levels than CC (mean difference: +6.46 ng/mL, *p* < 0.001). *CYP2R1* rs10741657 was also significant (F(2, 227) = 23.78, *p* < 0.001, η^2^ = 0.17), with GG carriers having the highest levels. *GC* rs2282679 showed no significant differences (*p* = 0.19, η^2^ = 0.02).

### 3.5. 25(OH)D) Across BMI Categories

An overall ANOVA revealed a statistically significant difference in serum 25(OH)D levels across BMI categories (*p* < 0.001). Tukey’s HSD post hoc analysis confirmed that normal-weight individuals had significantly higher 25(OH)D concentrations compared to all other groups (all *p* < 0.001), while no significant differences were observed among overweight and obese subgroups (*p* > 0.05).

Bonferroni-adjusted pairwise comparisons are detailed in Supplementary Table S3.

**Figure 3:**
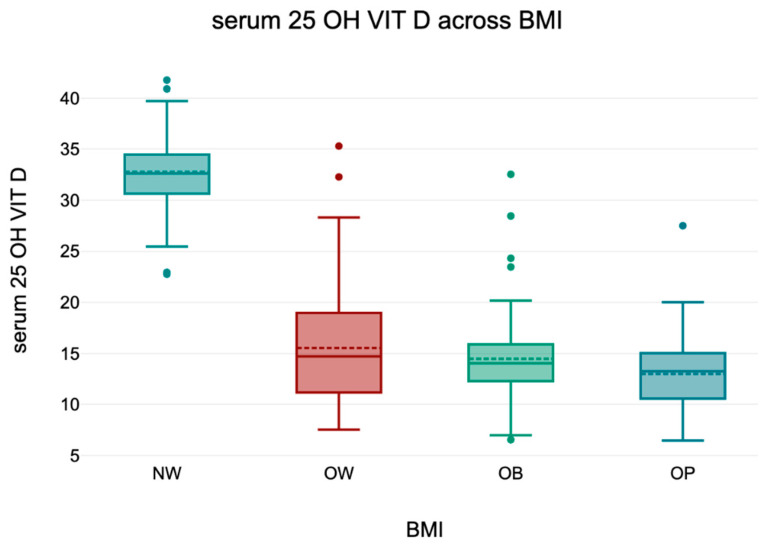
Serum 25(OH)D levels across BMI categories. Boxplot representation of serum 25(OH)D concentrations in relation to BMI categories. ANOVA followed by Tukey’s HSD test revealed significantly higher levels in normal-weight individuals compared to overweight and obese participants (all *p* < 0.001). No significant differences were found between OW and OB and OP (*p* > 0.05).

These findings align with the well-established inverse relationship between BMI and vitamin D status. Descriptive statistics are presented in Table 5 and a boxplot representation is shown in Figure 3.

To illustrate these differences visually, Figure 3 presents a graphical representation of serum 25(OH)D levels across BMI categories. The data highlight a progressive decline in vitamin D levels with increasing BMI, with the most pronounced drop observed in obese individuals.

### 3.6. Serum 25(OH)D Levels and Dietary Vitamin D Intake

Serum 25(OH)D levels and dietary vitamin D intake (as estimated by FFQ and measured by FFD) are presented in Table 6. While obese individuals consistently presented with lower serum 25(OH)D levels, their reported vitamin D intake did not differ substantially across BMI categories. However, FFQ values were on average 35 IU/day higher than FFD values, suggesting potential overestimation in retrospective reports.

To harmonize the intake data, we calculated a total vitamin D intake (IU/day) as the mean of FFQ and FFD values for each participant, capturing both habitual patterns and precise short-term intake. This integrative approach has been used in prior dietary studies to mitigate recall and reporting biases [39].

**Figure 4:**
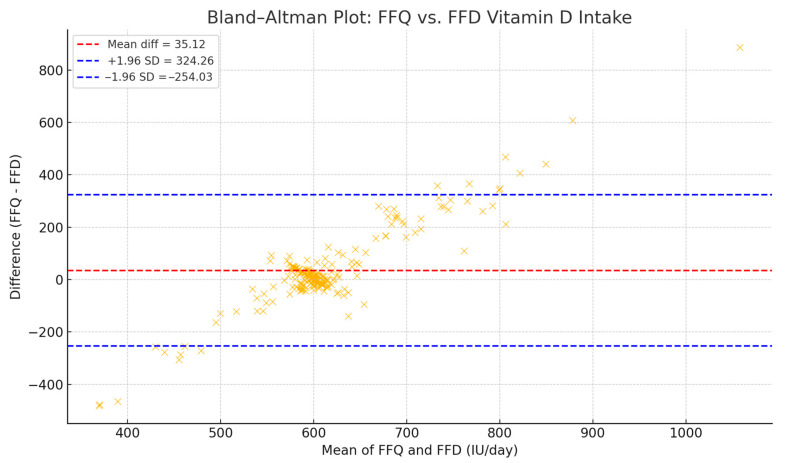
Bland–Altman agreement plot comparing FFQ and FFD estimates of daily vitamin D intake. The mean difference was 35.12 IU/day, with limits of agreement ranging from −254.03 to 324.26 IU/day.

To validate agreement between methods, a Bland–Altman analysis was performed (Figure 4), revealing a mean difference of +35.12 IU/day (FFQ − FFD), with 95% limits of agreement from −254.03 to +324.26 IU/day. These findings support the use of the harmonized value in population-level dietary assessments [40].

A one-way ANOVA confirmed a significant difference in serum 25(OH)D levels across BMI groups (F(3, 226) = 33.99, *p* < 0.001, partial η^2^ = 0.31), reinforcing the link between a higher BMI and lower vitamin D levels. Additionally, a significant interaction was observed between BMI group and measurement method (serum, FFQ, FFD) (F (6, 452) = 30.25, *p* < 0.001, partial η^2^ = 0.29), indicating that the effect of BMI on vitamin D status varies depending on the assessment method.

Post hoc Bonferroni tests revealed that vitamin D levels differed significantly between NW vs. OW, NW vs. OB, and NW vs. OP groups (*p* < 0.001). The difference between OW and OB was borderline significant (*p* = 0.08), while the comparison between OW and OP reached statistical significance (*p* = 0.01). No significant difference was observed between OB and OP (*p* = 1.00), suggesting that the greatest decline in serum 25(OH)D occurs between normal-weight and overweight groups, with further reductions being less pronounced among obese individuals.

A detailed summary of Bonferroni-adjusted pairwise comparisons—including mean differences, t-values, and *p*-values—for serum 25(OH)D across BMI groups is available in Supplementary Table S3.

Table 7 shows the summary of the one-way ANOVA results for serum 25(OH)D, FFQ, and FFD across BMI groups.

**Figure 5:**
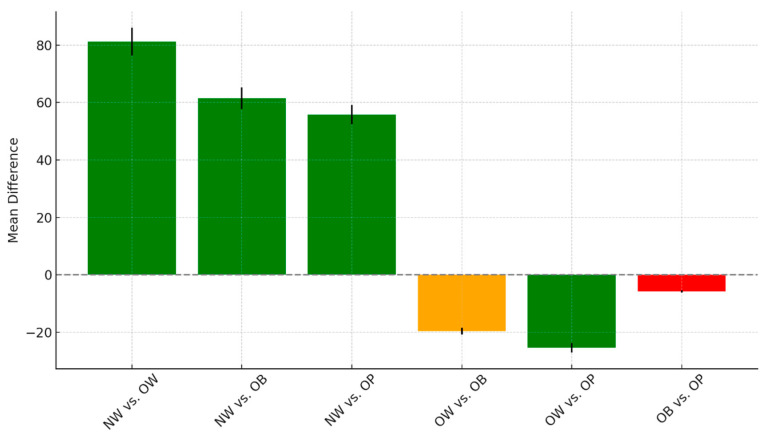
Pairwise group comparisons with mean difference and t-value error bars.

The pairwise comparisons among the different weight groups, including NW, OW, OB, and OP, reveal notable patterns in mean differences and statistical significance, as seen in Figure 5.

There were highly significant differences when comparing the normal-weight group to all other categories. Specifically, NW individuals showed a mean difference of 81.2 compared to the OW group (t = 9.74, *p* < 0.001), 61.53 compared to the OB group (t = 7.46, *p* < 0.001), and 55.72 compared to the OP group (t = 6.68, *p* < 0.001). These findings suggest consistently higher or lower values in the NW group relative to the others, with all comparisons achieving strong statistical significance. When comparing overweight to obese individuals, the mean difference was −19.67, with a t-value of −2.49 and a *p*-value of 0.08. This result approached but did not meet the conventional threshold for significance and was classified as borderline. In contrast, the comparison between the OW and OP groups showed a significant mean difference of −25.47 (t = −3.19, *p* = 0.01), indicating that these two groups differ meaningfully on the measured outcome. Finally, no significant difference was observed between the OB and OP groups, with a small mean difference of −5.8 (t = −0.74, *p* = 1.00). This suggests that these two categories are statistically similar in terms of the outcome variable analyzed. Overall, the NW group stands out as distinct from all others, and OW individuals differ significantly from OP, while OB and OP remain statistically comparable.

The figure shows mean differences and t-values for pairwise comparisons of serum 25(OH)D levels between BMI categories.

#### 3.6.1. Distribution of Serum hs-CRP Levels

**Figure 6:**
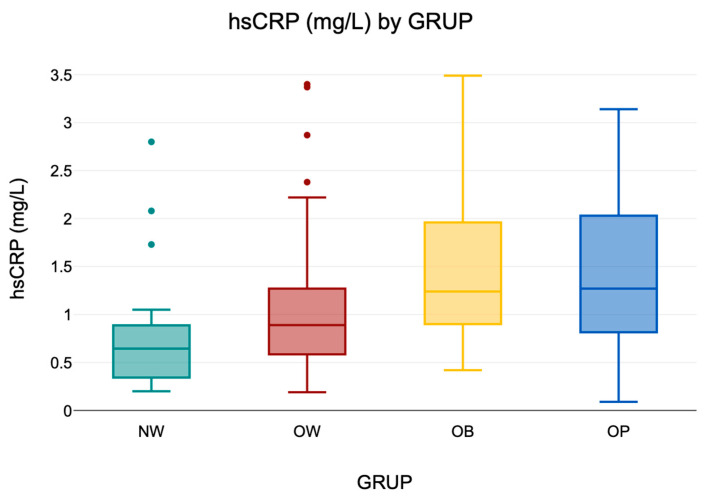
Boxplot of serum hs-CRP concentrations by BMI group (NW, OW, OB). The central line indicates the median; boxes represent the interquartile range (IQR); and whiskers denote the minimum and maximum values within 1.5× IQR. Outliers are shown as individual points where applicable.

Serum hs-CRP levels were analyzed as a proxy for low-grade systemic inflammation. In the overall cohort (*n* = 230), values ranged from 0.09 to 3.49 mg/L, indicating substantial interindividual variability. Descriptive values are presented in Table 8, and a visual summary is provided in Figure 6**,** illustrating the distribution of hs-CRP levels across BMI groups.

#### 3.6.2. Correlations Between hs-CRP and Serum 25(OH)D

An inverse correlation was observed between serum 25(OH)D and hs-CRP levels (ρ = −0.224, *p* < 0.001), suggesting that a lower vitamin D status is associated with higher systemic inflammation. This inverse relationship underscores the immunomodulatory potential of vitamin D. Descriptive statistics for these biomarkers are presented in Table 9.

**Figure 7:**
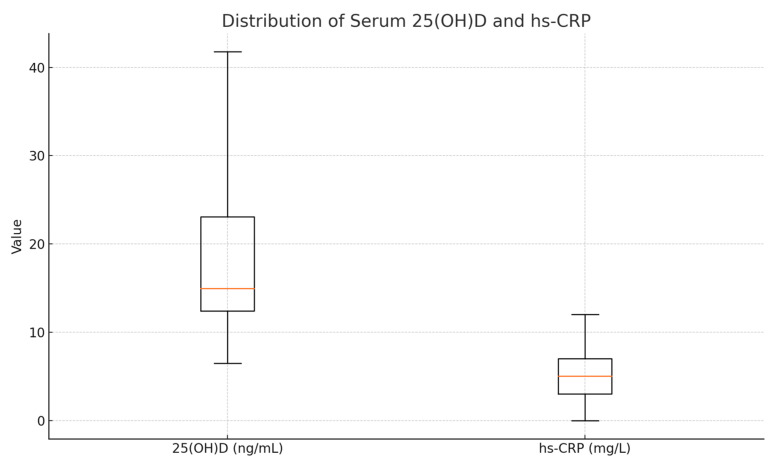
Boxplots of serum 25(OH)D (left) and hs-CRP (right). The box spans Q1–Q3 (mean ± SD), the orange line marks the median (here set equal to the mean), and whiskers extend to the true minimum and maximum. Units are ng/mL for 25(OH)D and mg/L for hs-CRP.

The relationship is illustrated in Figure 7, showing a negative trend between serum vitamin D and hs-CRP. When stratifying participants by 25(OH)D quartiles, a consistent decrease in hs-CRP was noted, supporting the potential anti-inflammatory role of vitamin D.

**Figure 8:**
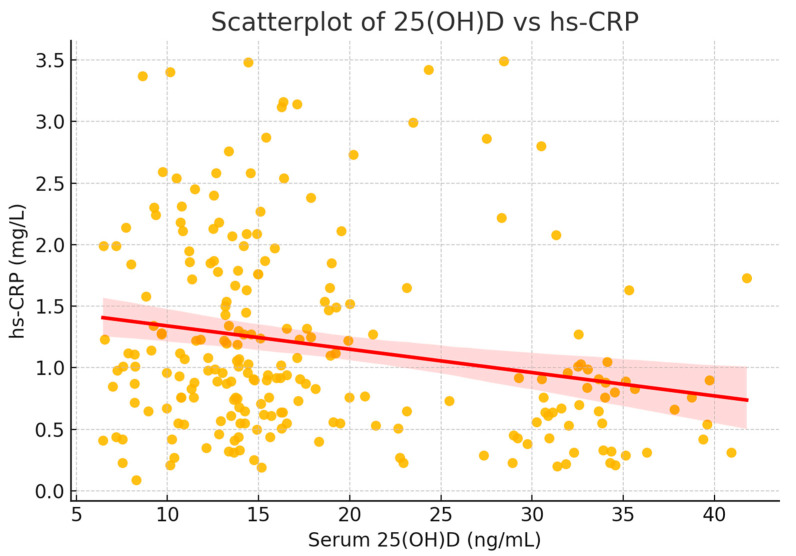
Scatterplot of serum 25(OH)D versus hs-CRP. The fitted regression line (red) with 95% confidence interval (shaded) shows a significant inverse association between vitamin D status and systemic inflammation (ρ = −0.224, *p* < 0.001).

To visualize this relationship, a scatterplot was generated, including a fitted regression line with 95% confidence interval (Figure 8). The plot illustrates the significant inverse correlation between vitamin D status and systemic inflammation.

To further explore this association, participants were stratified into quartiles based on serum 25(OH)D levels. hs-CRP levels decreased progressively across quartiles, with the lowest inflammatory markers observed in individuals with the highest vitamin D concentrations (Figure 8).

This consistent trend supports the hypothesis that an adequate vitamin D status may exert a protective anti-inflammatory effect. Altogether, vitamin D insufficiency and excess adiposity appear to act synergistically to elevate hs-CRP in this adult population.

#### 3.6.3. Correlations Between hs-CRP, VDR, CYP2R1, and GC Genotypes

To explore whether common vitamin-D-pathway variants modulate low-grade inflammation, we analyzed serum hs-CRP in relation to *VDR* rs731236, *CYP2R1* rs10741657, and *GC* rs2282679 genotypes (*n* = 230). Two complementary approaches were applied: Spearman’s rank correlation between hs-CRP (continuous) and the ordinal genotype code (0 = major-allele homozygote, 1 = heterozygote, 2 = minor-allele homozygote). The Kruskal–Wallis H test was used to compare hs-CRP distributions across the three genotype classes for each SNP (Table 10).

**Figure 9:**
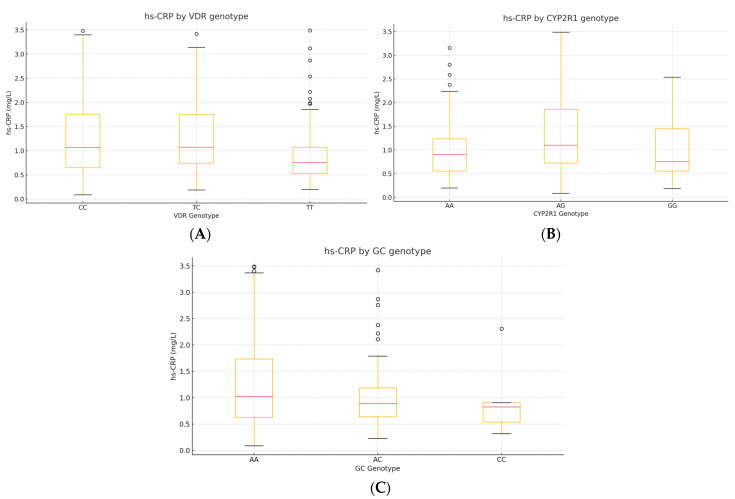
(**A**–**C**). Box-and-whisker plots of hs-CRP by genotype. (**A**) *VDR* rs731236, (**B**) *CYP2R1* rs10741657, and (**C**) *GC* rs2282679.

The plots depict the median (red line), interquartile range (box), and outliers. A step-wise increase in hs-CRP is apparent for carriers of the C allele of VDR (TC/CC)**,** while CYP2R1 AG heterozygotes display the highest median hs-CRP. No clear gradient is evident across GC genotypes (Figure 9).

### 3.7. Correlation and Regression Analyses

Pearson’s correlation analysis identified a significant negative correlation between BMI and serum 25(OH)D levels (r = −0.45, *p* < 0.01), further validating the inverse relationship observed in group comparisons.

A multiple linear regression analysis was conducted to assess the independent contributions of BMI, FFQ, and FFD to serum 25(OH)D levels. The overall model demonstrated a strong negative association between BMI and vitamin D levels, with obese groups exhibiting reductions of 16–19 ng/mL relative to the normal-weight group (*p* < 0.001). FFQ-estimated vitamin D intake had a modest positive effect (β = 0.01, *p* = 0.036), while FFD did not show a significant impact (*p* = 0.289). These results confirm that BMI remains the strongest predictor of vitamin D deficiency, with dietary intake playing a minor role (Table 11).

Further correlation analyses using Pearson’s and Spearman’s methods (Table 12 and Table 13) indicated a moderate positive correlation between serum 25(OH)D and FFQ-estimated vitamin D intake (r = 0.47, *p* < 0.001), whereas the correlation with FFD was not significant (r = −0.04, *p* = 0.542). As shown in Table 12, Pearson’s correlation revealed a significant positive relationship between FFQ-estimated vitamin D intake and serum 25(OH)D levels, while no such association was observed for FFD. Similarly, Spearman’s correlation (Table 13) confirmed the positive relationship between FFQ-estimated vitamin D intake and serum 25(OH)D, but again, FFD showed no significant correlation. These findings suggest that FFQ provides a reasonable estimate of vitamin D intake, whereas FFD does not correlate strongly with serum vitamin D levels.

Pearson correlation coefficients for serum 25(OH)D levels and vitamin D intake as estimated by the FFQ and FFD methods were calculated for the entire study population, without stratification by BMI groups. The *p*-value for the correlation between serum 25(OH)D and FFQ intake was <0.001, indicating a statistically significant relationship; Spearman’s correlation analysis was performed on the entire study population (without stratification by BMI groups) to evaluate the relationship between serum 25(OH)D levels and vitamin D intake estimated using the FFQ and FFD methods. The results are presented in Table 13.

Spearman correlation coefficients for serum 25(OH)D levels and vitamin D intake, as estimated by FFQ and FFD methods, were calculated for the entire study population (without stratification by BMI groups). The correlation between serum 25(OH)D and FFQ intake was statistically significant (*p* < 0.001), while no significant correlation was observed with FFD.

To explore genotype–phenotype relationships, one-way ANOVA analyses were conducted for VDR (rs731236), CYP2R1 (rs10741657), and GC (rs2282679) polymorphisms. Significant differences in serum 25(OH)D concentrations were observed by genotype for VDR (F = 15.34, *p* < 0.001) and CYP2R1 (F = 23.78, *p* < 0.001), with TT carriers of VDR displaying higher levels, and AG/GG genotypes of CYP2R1 associated with reduced 25(OH)D concentrations (see Supplementary Table S4 for details). No significant association was found for GC. These results support a genotype-dependent influence on vitamin D status.

## 4. Discussion

Our findings expand current understanding by demonstrating that specific vitamin D-related genotypes may influence both serum 25(OH)D levels and adiposity. Notably, the VDR rs731236 T-allele was associated with higher 25(OH)D concentrations and a lower BMI, suggesting a dual protective role, while the CYP2R1 rs10741657 A-allele was linked to both reduced vitamin D status and increased adiposity [41,42]), with potential long-term consequences for metabolic health, including an increased risk of conditions such as type 2 diabetes [43].

Obesity not only exacerbates vitamin D deficiency but may also affect vitamin D metabolism through genetic variations in the vitamin D receptor (VDR) gene. In our cohort, carriers of the TT genotype at rs731236 had significantly higher 25(OH)D levels compared to CC carriers (mean difference: +6.46 ng/mL, *p* < 0.001), indicating a genotype-dependent response that may further modulate the impact of adiposity on vitamin D bioavailability.

This finding suggests that the TT genotype may attenuate the negative impact of obesity on vitamin D bioavailability, potentially offering a protective mechanism in individuals with a higher BMI. These results support previous studies indicating that VDR polymorphisms influence not only vitamin D metabolism but also adiposity-related phenotypes, reinforcing the concept of a gene–environment interaction.

Given the central role of VDR in calcium homeostasis and immune regulation, such interactions may have broader clinical implications, particularly in the personalized management of vitamin D deficiency [44,45,46].

Additionally, obesity may impair vitamin D metabolism through mechanisms such as altered tissue distribution and volumetric dilution. These alterations are consistent with the findings of Norman et al. (2007), who highlighted the reduced bioavailability of vitamin D in individuals with obesity due to altered tissue distribution and metabolic dynamics [47,48,49].

Although estimated vitamin D intake, assessed by both FFQ and FFD, was comparable across BMI categories, serum 25(OH)D levels declined progressively with increasing adiposity. This suggests that reduced vitamin D status in overweight and obese individuals is not attributable to dietary intake, but more likely reflects the effects of adipose tissue sequestration and altered metabolism [46,49].

In our cohort, both FFQ and FFD estimates provided consistent patterns of vitamin D intake across BMI groups. While minor differences between methods are expected due to recall and reporting variability, the declining trend in serum 25(OH)D with increasing adiposity remained evident regardless of the dietary assessment tool. This supports the notion that reduced vitamin D status in overweight and obese individuals cannot be attributed solely to dietary intake.

The modest but statistically significant correlations between reported vitamin D intake and serum 25(OH)D levels support the contribution of diet but also highlight the dominant role of adiposity and possibly genetic factors in determining vitamin D bioavailability.

The positive association observed between BMI and serum hs-CRP levels in our study reinforces the well-established link between adiposity and chronic low-grade inflammation [47]. These findings are consistent with previous meta-analyses indicating that excess adipose tissue promotes systemic inflammation through the increased secretion of pro-inflammatory cytokines such as IL-6 and TNF-α.

The strong inverse correlation between serum 25(OH)D levels and hs-CRP further supports the anti-inflammatory role of vitamin D. An adequate vitamin D status may help mitigate the inflammatory effects of excess adiposity by modulating immune pathways. Mechanistically, vitamin D inhibits NF-κB activation and reduces hepatic CRP synthesis, thereby attenuating systemic [43,49].

Among the genetic variants analyzed, the *CYP2R1* rs10741657 AA genotype was significantly associated with elevated hs-CRP levels, suggesting a link between impaired vitamin D hydroxylation and systemic inflammation. Individuals with this genotype exhibited both lower 25(OH)D and higher inflammatory markers, reinforcing the hypothesis that reduced vitamin D bioavailability may amplify inflammatory pathways in obesity.

While *VDR* and *GC* polymorphisms did not show direct associations with hs-CRP, their influence on serum 25(OH)D may contribute indirectly to the observed inflammatory profile.

Limitations: This study has several limitations that warrant consideration. First, its cross-sectional design precludes any inference of causality between BMI and serum 25(OH)D levels. Second, although the sample is relevant to the study’s objectives, it may not fully capture the heterogeneity of the general population, potentially limiting the external validity of our findings.

While adjustments were made for key confounders, other influential factors—such as sun exposure, physical activity, and broader environmental variables—were not included in the main analyses and could contribute to the observed interindividual variability in vitamin D status. Although data on occupational setting and sunlight exposure were collected at baseline, these were excluded from the core statistical models to maintain a focus on the primary variables of interest: BMI, dietary intake, and genetic background.

Future Directions: Future research should aim to elucidate the causal pathways that link BMI, vitamin D metabolism, and genetic variability. Longitudinal studies conducted on larger, ethnically diverse cohorts are needed to confirm these findings and to better understand how genetic predisposition interacts with modifiable factors—such as sun exposure, physical activity, and dietary habits—in shaping vitamin D status.

Integrating multi-omics data and environmental exposures would enable more refined models of vitamin D homeostasis across metabolic phenotypes. In particular, mechanistic studies exploring how *CYP2R1* polymorphisms contribute to both vitamin D insufficiency and features of metabolic syndrome may reveal novel targets for intervention.

Such insights could support the development of genotype-informed strategies for preventing or mitigating vitamin D deficiency, especially in individuals at risk for obesity-related metabolic disorders.

## 5. Conclusions

This study highlights the complex interplay between genetic polymorphisms, adiposity, and vitamin D status. Our findings demonstrate that individuals carrying the VDR rs731236 T-allele exhibit both higher serum 25(OH)D concentrations and lower BMI, supporting a dual protective role. Conversely, the CYP2R1 rs10741657 A-allele was associated with vitamin D insufficiency, increased adiposity, and elevated hs-CRP levels, suggesting a genetic predisposition to both metabolic and inflammatory disturbances.

Importantly, serum 25(OH)D levels declined progressively with increasing BMI, independent of dietary intake, reinforcing the role of adiposity-related sequestration and altered metabolism. The inverse association between 25(OH)D and hs-CRP further supports the anti-inflammatory role of vitamin D and its potential relevance in obesity-associated low-grade inflammation. Together, these results emphasize the importance of integrating genetic, nutritional, and metabolic data in the evaluation and management of vitamin D deficiency. Genotype-informed approaches may enhance the precision of supplementation strategies, particularly in populations at risk for obesity and chronic inflammation.

## Figures and Tables

**Table 1:** Demographic and anthropometric characteristics of the studied groups.

Group	Average Age (Years)	Sex (Male/Female)	Origin (Urban/Rural)	Weight (kg) (Average Value)	Height (cm) (Average Value)	BMI (kg/m^2^) (Average Value)
NW (Normal Weight)	36	23/27	36/14 (72%/28%)	64.5	171	22
OW (Overweight)	38	25/34	44/15 (25.37/11.21%)	81	170	27.5
OB (Obese I)	40.5	13/49	43/19 (26.66%/11.78%)	89.5	169	32.45
OP (Obese II/III)	37	14/45	45/14 (26.55%/8.26%)	90	165	37

**Table 2:** Genotype distribution and allele frequencies for *VDR*, *CYP2R1*, and *GC* polymorphisms.

Gene	SNP	Genotype	Frequency (%)	χ^2^	*p*-Value	Biological Interpretation
*VDR*	rs731236	TT	31.3	7.59	0.022	Optimal 25(OH)D levels [34]
		CT	49.1			Intermediate vitamin D status
		CC	19.6			Increased risk of vitamin D deficiency
*CYP2R1*	rs10741657	AA	40.4	11.62	0.003	Efficient hepatic conversion of vitamin D [34]
		AG	33.5			Intermediate conversion efficiency
		GG	26.1			Inefficient hepatic conversion
*GC*	rs2282679	AA	58.3	2.39	0.42	Efficient vitamin D transport [26]
		AC	40.0			Partially impaired transport
		CC	1.7			Increased risk of vitamin D deficiency

Significant associations were observed for *VDR* rs731236 (*p* = 0.022) and *CYP2R1* rs10741657 (*p* = 0.003), while *GC* rs2282679 was not significant (*p* = 0.42). The biological interpretation reflects each genotype’s potential impact on vitamin D metabolism.

**Table 3:** BMI across VDR, CYP2R1, and GC genotypes.

SNP/Genotype	*n*	BMI (kg m^−2^) Mean ± SD	Median [Min–Max]	H (df)	*p*-Value	ε^2^
VDR rs731236				25.45 (2)	1.2 × 10^−6^	0.105
TT	72	28.46 ± 5.34	28.2 [18.8–42.6]			
TC	134	31.06 ± 6.02	31.6 [19.2–47.7]			
CC	22	33.14 ± 5.77	33.8 [21.1–43.8]			
CYP2R1 rs10741657				32.61 (2)	1.7 × 10^−7^	0.137
AA	58	33.51 ± 5.61	33.9 [22.7–46.4]			
AG	135	29.56 ± 5.85	28.4 [18.6–47.7]			
GG	35	30.02 ± 6.46	29.3 [18.8–45.2]			
GC rs2282679				4.44 (3)	0.18	0.006
AA	164	30.69 ± 6.11	31.2 [18.8–47.7]			
AC	57	29.40 ± 5.84	27.9 [18.6–46.2]			
CC	5	26.86 ± 6.73	24.6 [20.7–35.0]			

**Table 4:** Serum 25(OH)D concentrations according to VDR, CYP2R1, and GC genotypes.

SNP/Genotype	*n*	Mean ± SD (ng mL^−1^)	Median [min–max]	F (df)	*p*-Value	η^2^p
VDR rs731236				15.34 (2, 227)	<0.001	0.12
TT	72	34.6 ± 7.8	34.2 [16.3–53.1]			
TC	134	29.4 ± 8.1	28.6 [10.4–55.0]			
CC	22	28.1 ± 7.5	27.8 [12.1–45.9]			
CYP2R1 rs10741657				23.78 (2, 227)	<0.001	0.17
AA	58	26.9 ± 7.4	26.2 [11.5–45.1]			
AG	135	32.7 ± 7.9	32.5 [14.2–55.0]			
GG	35	34.0 ± 7.3	33.8 [18.4–50.7]			
GC rs2282679				1.59 (3, 226)	0.19	0.02
AA	164	30.8 ± 8.0	30.1 [11.5–55.0]			
AC	57	31.2 ± 7.4	31.0 [14.2–49.6]			
CC	5	29.6 ± 6.8	29.4 [21.3–38.6]			

**Table 5:** Descriptive statistics for serum 25(OH)D levels across BMI groups.

Group	Mean (Serum 25(OH)D) (ng/mL)	Std. Deviation (ng/mL)	Minimum (ng/mL)	Maximum (ng/mL)	Quartile 1 (ng/mL)	Quartile 3 (ng/mL)
NW	32.78	4.00	22.76	41.77	30.65	34.49
OW	15.53	5.81	7.54	35.31	11.17	18.98
OB	14.47	4.54	6.55	32.54	12.3	15.89
OP	13.00	3.79	6.47	27.51	10.57	15.03

Higher BMI was significantly associated with lower serum vitamin D levels (*p* < 0.001).

**Table 6:** Mean Serum 25(OH)D levels and vitamin D intake by BMI group.

Group	Serum 25(OH)D (ng/mL)	FFQ Vitamin D Intake (IU/day)	FFD Vitamin D Intake (IU/day)	Total Vitamin D Intake (IU/day)
NW	32.78	772.61	594.70	466.70
OW	15.53	542.78	598.19	385.50
OB	14.47	605.62	595.42	405.17
OP	13.00	625.36	594.56	410.97
Overall Mean	18.35	630.87	595.75	414.99

The “Overall Mean” represents the combined mean values for the entire study sample (*n* = 230), providing a general benchmark for vitamin D status and dietary intake.

**Table 7:** Summary of one-way ANOVA results for serum 25(OH)D, FFQ, and FFD across BMI groups.

Analysis Component	F Value	df	*p*-Value	Partial η^2^
Serum 25(OH)D, FFQ, FFD (Main Effect)	5243.07	(2, 226)	<0.001	0.91
BMI Group (Main Effect)	33.99	(3, 226)	<0.001	0.31
Interaction (BMI × Measurement Method)	30.25	(6, 452)	<0.001	0.29

Where F Value represents the F statistic, which quantifies the differences between group means; df (Degrees of Freedom) indicates the number of independent values used in the calculation; *p*-value is the probability that the observed result is due to chance; values below 0.05 denote statistical significance. Partial η^2^ is the proportion of the total variance that is attributable to the factor being tested, reflecting the effect size.

**Table 8:** Serum hs-CRP concentrations across BMI categories.

BMI Group	*n*	Mean (mg/L)	Std. Deviation	Minimum	Maximum
NW	50	0.70	0.48	0.20	2.80
OW	59	1.05	0.71	0.19	3.40
OB	62	1.46	0.81	0.42	3.49
OP	59	1.43	0.80	0.09	3.14

Values represent serum hs-CRP levels (mg/L) expressed as mean ± standard deviation. BMI categories. Higher BMI was associated with elevated inflammatory status, as reflected by increased hs-CRP concentrations.

**Table 9:** Descriptive statistics for serum hs-CRP and 25(OH)D.

Variable	Mean	SD	Minimum	Maximum
hs-CRP (mg/L)	1.18	0.78	0.09	3.49
25(OH)D (ng/mL)	18.35	8.95	6.47	41.77

Data are reported as mean, standard deviation (SD), minimum, and maximum values. Units: hs-CRP in mg/L; 25(OH)D in ng/mL.

**Table 10:** Association between vitamin-D-related genotypes and hs-CRP.

Gene (SNP)	Spearman ρ	*p* (Spearman)	Kruskal–Wallis *H*	*p* (KW)
VDR (rs731236)	0.175	0.008	9.52	0.009
CYP2R1 (rs10741657)	0.065	0.329	9.61	0.008
GC (rs2282679)	0.072	0.280	2.50	0.286

*VDR* rs731236 and *CYP2R1* rs10741657 showed statistically significant associations with hs-CRP, supporting a genotype-dependent inflammatory profile in individuals with low vitamin D activity. *GC* rs2282679 was not associated with hs-CRP in this cohort.

**Table 11:** Multiple linear regression analysis predicting serum 25(OH)D level.

Predictor	Unstandardized Coefficient (B)	Standardized Coefficient (β)	Standard Error (SE)	t	*p*-Value
Constant	35.41	–	6.41	5.52	<0.001
OW	−16.00	−0.78	1.06	−15.16	<0.001
OB	−17.42	−0.87	0.97	−18.00	<0.001
OP	−19.01	−0.93	0.96	−19.89	<0.001
FFQ Vitamin D Intake (IU/day)	0.01	0.09	0.01	2.11	0.036
FFD Vitamin D Intake (IU/day)	−0.01	−0.04	0.01	−1.06	0.289

Results indicate significant negative associations for higher BMI categories (OW, OB, OP) with serum 25(OH)D, and a minor positive effect of FFQ-estimated vitamin D intake. B = unstandardized coefficient; β = standardized coefficient; SE = standard error; t = t-value; *p* = *p*-value.

**Table 12:** Pearson correlation coefficients were calculated for all study participants to assess the relationship between serum 25(OH)D levels and vitamin D intake estimated through FFQ and FFD methods.

Measurement Methods	Serum 25(OH)D	FFQ Vitamin D Intake (IU/day)	FFD Vitamin D Intake (IU/day)
Serum 25(OH)D	1	0.47	−0.04
FFQ Vitamin D Intake (IU/day)	0.47	1	0.07
FFD Vitamin D Intake (IU/day)	−0.04	0.07	1

**Table 13:** Spearman correlation coefficients between serum 25(OH)D levels and vitamin D intake estimated by FFQ and FFD methods in the entire study sample.

Measurement Methods	Serum 25(OH)D	FFQ Vitamin D Intake (IU/day)	FFD Vitamin D Intake (IU/day)
Serum 25(OH)D	1	0.35	−0.13
FFQ Vitamin D Intake (IU/day)	0.35	1	−0.01
FFD Vitamin D Intake (IU/day)	−0.13	−0.01	1

## Data Availability

All the data processed in this article are part of the research for a doctoral thesis, being archived in the medical office, where the interventions were performed.

## Supplementary Materials

The following supporting information can be downloaded at: https://www.mdpi.com/article/10.3390/diseases13070219/s1, Table S1: Post-hoc comparisons (adjusted *p*-values) for; Table S2: One-way ANOVA results evaluating the associations between serum 25(OH)D concentrations and genetic variants in *VDR*, *CYP2R1*, and *GC*. Includes sum of squares, degrees of freedom, F statistic and *p*-values; Table S3: Bonferroni-Adjusted Pairwise Comparisons Between BMI Groups for Serum 25(OH)D Levels; Table S4. Serum 25(OH)D levels (ng/mL) by genotype for VDR, CYP2R1 and GC polymorphisms (one-way ANOVA).

## References

[1] Cui A., Zhang T., Xiao P., Fan Z., Wang H., Zhuang Y. (2023). Global and regional prevalence of vitamin D deficiency in population-based studies from 2000 to 2022: A pooled analysis of 7.9 million participants. Front. Nutr..

[2] Chirita-Emandi A., Socolov D., Haivas C., Calapiș A., Gheorghiu C., Puiu M. (2015). Vitamin D status: A different story in the very young versus the very old Romanian patients. PLoS ONE.

[3] Ministerul Sanatatii C.N.D.A. (2019). Raport de Activitate. Ghidul Pentru Evaluarea Statusului Vitaminei D la Adulți.

[4] Sahay M., Sahay R. (2012). Rickets–vitamin D deficiency and dependency. IJEM.

[5] De Martinis M., Allegra A., Sirufo M.M., Tonacci A., Pioggia G., Raggiunti M., Ginaldi L., Gangemi S. (2021). Vitamin D Deficiency, Osteoporosis and Effect on Autoimmune Diseases and Hematopoiesis: A Review. Int. J. Mol. Sci..

[6] Haider F., Ghafoor H., Hassan O.F., Farooqui K., Bel Khair A.O.M., Shoaib F. (2023). Vitamin D and Cardiovascular Diseases: An Update. Cureus.

[7] Alzohily B., AlMenhali A., Gariballa S., Munawar N., Yasin J., Shah I. (2024). Unraveling the complex interplay between obesity and vitamin D metabolism. Sci. Rep..

[8] Roxana V.-R., Guadarrama-Lopez A.L., Martinez-Carrillo B.E., Benitez-Arciniega A.D. (2015). Vitamins and Type 2 Diabetes Mellitus. EMID-DT.

[9] Ogeyingbo O.D., Ahmed R., Gyawali M., Venkatesan N., Bhandari R., Botleroo R.A., Kareem R., Elshaikh A.O. (2021). The Relationship Between Vitamin D and Asthma Exacerbation. Cureus.

[10] Flønes I.H., Fernandez-Vizarra E., Lykouri M., Brakedal B., Skeie G.O., Miletic H., Lilleng P.K., Alves G., Tysnes O.-B., Haugarvoll K. (2018). Neuronal complex I deficiency occurs throughout the Parkinson’s disease brain, but is not associated with neurodegeneration or mitochondrial DNA damage. Acta Neuropathol..

[11] Schmid A., Walther B. (2013). Natural vitamin D content in animal products. Adv. Nutr..

[12] Khan M.U., Gautam G., Jan B., Zahiruddin S., Parveen R., Ahmad S. (2022). Vitamin D from Vegetable VV Sources: Hope for the Future. Phytomed. Plus.

[13] Kühn J., Schröter A., Hartmann B.M., Stangl G.I. (2018). Cocoa and chocolate are sources of vitamin D(2). Food Chem..

[14] Aiello G., Lombardo M., Baldelli S. (2024). Exploring Vitamin D Synthesis and Function in Cardiovascular Health: A Narrative Review. Appl. Sci..

[15] Maurya V.K., Aggarwal M. (2017). Factors influencing the absorption of vitamin D in GIT: An overview. J. Food Sci. Technol..

[16] Holick M.F. (2011). Vitamin D: Evolutionary, physiological and health perspectives. Curr. Drug Targets.

[17] Garcia M., Seelaender M., Sotiropoulos A., Coletti D., Lancha A.H. (2019). Vitamin D, muscle recovery, sarcopenia, cachexia, and muscle atrophy. Nutrition.

[18] Stroia C.M., Ghitea T.C., Vrânceanu M., Mureșan M., Bimbo-Szuhai E., Pallag C.R., Pallag A. (2024). Relationship between Vitamin D3 Deficiency, Metabolic Syndrome and VDR, GC, and CYP2R1 Gene Polymorphisms. Nutrients.

[19] Trifan D.F., Tirla A.G., Moldovan A.F., Moș C., Bodog F., Maghiar T.T., Manole F., Ghitea T.C. (2023). Can Vitamin D Levels Alter the Effectiveness of Short-Term Facelift Interventions?. Healthcare.

[20] Trifan D.F., Tirla A.G., Mos C., Danciu A., Bodog F., Manole F., Ghitea T.C. (2023). Involvement of Vitamin D3 in the Aging Process According to Sex. Cosmetics.

[21] Wang Y., Zhu J., DeLuca H.F. (2012). Where is the vitamin D receptor?. Arch. Biochem. Biophys..

[22] Bahrami A., Sadeghnia H.R., Tabatabaeizadeh S.A., Bahrami-Taghanaki H., Behboodi N., Esmaeili H., Ferns G.A., Mobarhan M.G., Avan A. (2018). Genetic and epigenetic factors influencing vitamin D status. J. Cell Physiol..

[23] Manousaki D., Mitchell R., Dudding T., Haworth S., Harroud A., Forgetta V., Shah R.L., Luan J., Langenberg C., Timpson N.J. (2020). Genome-wide Association Study for Vitamin D Levels Reveals 69 Independent Loci. Am. J. Hum. Genet..

[24] Hyppönen E., Vimaleswaran K.S., Zhou A. (2022). Genetic Determinants of 25-Hydroxyvitamin D Concentrations and Their Relevance to Public Health. Nutrients.

[25] Voltan G., Cannito M., Ferrarese M., Ceccato F., Camozzi V. (2023). Vitamin D: An Overview of Gene Regulation, Ranging from Metabolism to Genomic Effects. Genes.

[26] Asghari G., Yuzbashian E., Najd-Hassan-Bonab L., Mirmiran P., Daneshpour M.S., Azizi F. (2023). Association of rs2282679 polymorphism in vitamin D binding protein gene (GC) with the risk of vitamin D deficiency in an iranian population: Season-specific vitamin D status. BMC Endocr. Disord..

[27] Forster H., Fallaize R., Gallagher C., O’Donovan C.B., Woolhead C., Walsh M.C., Macready A.L., Lovegrove J.A., Mathers J.C., Gibney M.J. (2014). Online dietary intake estimation: The Food4Me food frequency questionnaire. J. Med. Internet Res..

[28] De Giuseppe R., Tomasinelli C.E., Cena H., Braschi V., Giampieri F., Preatoni G., Centofanti D., Princis M.P., Bartoletti E., Biino G. (2022). Development of a Short Questionnaire for the Screening for Vitamin D Deficiency in Italian Adults: The EVIDENCe-Q Project. Nutrients.

[29] Lips P., Cashman K.D., Lamberg-Allardt C., Bischoff-Ferrari H.A., Obermayer-Pietsch B., Bianchi M.L., Stepan J., El-Hajj Fuleihan G., Bouillon R. (2019). Current vitamin D status in European and Middle East countries and strategies to prevent vitamin D deficiency: A position statement of the European Calcified Tissue Society. Eur. J. Endocrinol..

[30] Yahya Rayat D. (2024). Longitudinal Monitoring of Physiological, Psychological and Molecular Responses to Different Dietary Interventions: A Precision Health Approach. Master’s Thesis.

[31] Musgrave K.O., Giambalvo L., Leclerc H.L., Cook R.A., Rosen C.J. (1989). Validation of a quantitative food frequency questionnaire for rapid assessment of dietary calcium intake1. J. Am. Diet Assoc..

[32] Xu L., Porteous J.E., Phillips M.R., Zheng S. (2000). Development and Validation of a Calcium Intake Questionnaire for Postmenopausal Women in China. Ann. Epidemiol..

[33] Dietary Assessment Primer National Institutes of Health, National Cancer Institute. https://dietassessmentprimer.cancer.gov/.

[34] EFSA Panel on Dietetic Products, Nutrition and Allergies (2016). Dietary reference values for vitamin D. EFSA J..

[35] The Office of Dietary Supplements (ODS) of the National Institutes of Health (NIH) (2023) Fact Sheet for Health Professionals—Vitamin D. https://www.scirp.org/reference/referencespapers?referenceid=3553134.

[36] Saliva DNA Extraction & WGA Amplification Protocol. https://www.sigmaaldrich.com/RO/en/technical-documents/protocol/genomics/dna-and-rna-purification/extraction-of-dna-from-saliva.

[37] National Institutes of Health Office of Dietary Supplements Vitamin D-Fact Sheet for Health Professionals. https://www.scirp.org/reference/referencespapers?referenceid=2356707.

[38] Spencer L.R., Rollo M., Hauck Y., Macdonald-Wicks L., Wood L., Hutchesson M., Giglia R., Smith R., Collins C. (2015). The effect of weight management interventions that include a diet component on weight-related outcomes in pregnant and postpartum women: A systematic review protocol. JBI Database Syst. Rev. Implement. Rep..

[39] Nuti R., Gennari L., Cavati G., Pirrotta F., Gonnelli S., Caffarelli C., Tei L., Merlotti D. (2023). Dietary vitamin d intake in italian subjects: Validation of a frequency food questionnaire (ffq). Nutrients.

[40] Bishop K.J., Wilmer C.E., Soh S., Grzybowski B.A. (2009). Nanoscale forces and their uses in self-assembly. Small.

[41] Araújo E., Lima S., Galdino O.A., Arrais R.F., de Souza K.S.C., de Rezende A.A. (2022). Association of CYP2R1 and VDR Polymorphisms with Metabolic Syndrome Components in Non-Diabetic Brazilian Adolescents. Nutrients.

[42] Cheng S., Massaro J.M., Fox C.S., Larson M.G., Keyes M.J., McCabe E.L., Robins S.J., O’Donnell C.J., Hoffmann U., Jacques P.F. (2010). Adiposity, cardiometabolic risk, and vitamin D status: The Framingham Heart Study. Diabetes.

[43] Vranić L., Mikolašević I., Milić S. (2019). Vitamin D Deficiency: Consequence or Cause of Obesity?. Medicina.

[44] Alrushaid S., Davies N.M., Martinez S.E., Sayre C.L. (2017). Stereospecific pharmacokinetic characterization of liquiritigenin in the rat. Res. Pharm. Sci..

[45] Gawin F.H., Kleber H.D. (1988). Evolving conceptualizations of cocaine dependence. Yale J. Biol. Med..

[46] Colaneri M., Di Benedetto A., Marvulli L.N., Bocchio F., Cutti S., Marena C., Calvi M., Bruno R. (2022). Are people previously infected with SARS-CoV-2 likely to experience COVID-19 symptoms again after vaccination? Results from an Italian COVID-19 referral center. Hum. Vaccin. Immunother..

[47] Kim S.J., Sohn Y.B., Cho S.-Y., Choi Y.O., Kim C.H., Jin D.-K. (2013). Serum levels of FGF21 are reduced and negatively correlated with adiponectin in children with Prader-Willi syndrome. Int. J. Pediatr. Endocrinol..

[48] Sadeghi M., Ordway B., Rafiei I., Borad P., Fang B., Koomen J.L., Zhang C., Yoder S., Johnson J., Damaghi M. (2020). Integrative Analysis of Breast Cancer Cells Reveals an Epithelial-Mesenchymal Transition Role in Adaptation to Acidic Microenvironment. Front Oncol.

[49] Norman A.W. (2008). From vitamin D to hormone D: Fundamentals of the vitamin D endocrine system essential for good health. Am. J. Clin. Nutr..

