# Supplementary material for: The Association of VDR, CYP2R1, and GC Gene Polymorphisms, Dietary Intake, and BMI in Regulating Vitamin D Status
Source: Diseases. 2025 Jul 14;13(7):219. doi: 10.3390/diseases13070219 (PMC12293828; doi:10.3390/diseases13070219)
Supplement: Supplementary file 1 [file diseases-13-00219-s001.zip › diseases-3702449-supplementary.pdf]

Supplimentary Materials

BMI across genotypes of *VDR* rs731236 and *CYP2R1* rs10741657.

Differences in BMI (Δ) between genotypic groups were calculated after significant Kruskal–Wallis results. Adjusted p-values were obtained using Dunn's test with Bonferroni correction.

Effect-size (ε^2) ≥ 0.10 was considered moderate.

Table S1. Post-hoc comparisons (adjusted p-values) for.

SNP	Comparison	Δ BMI (kg m^{-2})	*p*-adj
**VDR**	TT vs TC	–2.60	3.1×10^{-5}
	TT vs CC	–4.68	1.2×10^{-6}
	TC vs CC	–2.08	0.041
**CYP2R1**	AA vs AG	+3.95	3.2×10^{-7}
	AA vs GG	+3.49	0.016
	AG vs GG	–0.46	0.0011

ε^2 = effect-size estimate for Kruskal–Wallis; values ≥0.10 denote moderate effect.

Table S2. One-way ANOVA results evaluating the associations between serum 25(OH)D concentrations and genetic variants in *VDR, CYP2R1*, and *GC*. Includes sum of squares, degrees of freedom, F statistic and p-values.

Unnamed: 0	Unnamed: 1	sum_sq	df	F	PR(>F)
Vitamin D vs VDR	C(VDR)	2181.922	2.0	15.336	0.0
Vitamin D vs VDR	Residual	16148.477	227.0	nan	nan
Vitamin D vs CYP2R1	C(CYP2R1)	3175.436	2.0	23.782	0.0
Vitamin D vs CYP2R1	Residual	15154.963	227.0	nan	nan
Vitamin D vs GC	C(GC)	380.277	3.0	1.596	0.191
Vitamin D vs GC	Residual	17950.122	226.0	nan	nan

One-way ANOVA results evaluating the associations between serum 25(OH)D concentrations and genetic variants in VDR (rs731236), CYP2R1 (rs10741657), and GC (rs2282679). The model included the F statistic, p-value, and degrees of freedom. Significant associations were observed for VDR and CYP2R1, but not for GC.

Table S3. Bonferroni-Adjusted Pairwise Comparisons Between BMI Groups for Serum 25(OH)D Levels.

Comparison	Mean Difference	t-value	p-value
NW vs OW	81.20	9.74	<0.001
NW vs OB	61.53	7.46	<0.001
NW vs OP	55.72	6.68	<0.001
OW vs OB	-19.67	-2.49	0.080
OW vs OP	-25.47	-3.19	0.010
OB vs OP	-5.80	-0.74	1.000

Post-hoc Bonferroni-adjusted pairwise comparisons between BMI categories for serum 25(OH)D levels. The table presents the mean difference, standard error (SE), t-values, and exact p-values for

each comparison. These detailed results complement the main ANOVA findings by clarifying specific group contrasts and their statistical significance, thereby enhancing the interpretability of BMI-related differences in vitamin D status.

Table S4. Serum 25(OH)D levels (ng/mL) by genotype for VDR, CYP2R1 and GC polymorphisms (one-way ANOVA)**.**

SNP (Genotype)	n	Mean ± SD	Median [Min–Max]	F(df)	p-value
**VDR rs731236**				**15.34 (2, 227)**	**< 0.001**
– CC	45	14.11 ± 5.03	13.91 [7.26–32.54]		
– TC	113	17.37 ± 8.59	14.42 [6.47–41.77]		
– TT	72	22.54 ± 9.76	21.34 [7.19–39.72]		
**CYP2R1 rs10741657**				**23.78 (2, 227)**	**< 0.001**
– AA	81	24.24 ± 9.53	23.88 [6.47–41.77]		
– AG	122	17.56 ± 7.53	15.15 [7.19–38.67]		
– GG	27	13.84 ± 5.91	12.47 [6.75–29.35]		
**GC rs2282679**				**1.86 (2, 227)**	0.158
– AA	117	18.52 ± 8.87	16.32 [6.47–41.77]		
– AC	94	17.06 ± 7.77	14.21 [6.75–39.72]		
– CC	19	15.91 ± 6.85	15.05 [7.19–33.16]		

Data are expressed as means ± SD and median [min–max]. One-way ANOVA was used to compare serum 25(OH)D levels across genotypes. Significant associations were found for VDR and CYP2R1, but not for GC.
